# Comparison of Cold-Cup Biopsy Versus Resection Biopsy in the Early Detection of Detrusor Muscle Invasion in the Case of Bladder Tumor

**DOI:** 10.7759/cureus.94420

**Published:** 2025-10-12

**Authors:** Saifullah Khan, Faiza Hayat, Qudratullah Wazir, Sania Gul

**Affiliations:** 1 Department of Urology, Institute of Kidney Diseases Peshawar, Peshawar, PAK; 2 Artificial Intelligence in Healthcare, Intelligent Information Processing Lab, National Centre of Artificial Intelligence, NED University of Engineering and Technology (UET), Peshawar, PAK

**Keywords:** bladder cancer, cold-cup biopsy, detrusor muscle invasion, resection biopsy, transurethral resection of bladder tumor (turbt)

## Abstract

Bladder cancer continues to represent a major global health burden, and accurate early staging is crucial for planning an appropriate treatment strategy regarding the presence of detrusor muscle (DM) invasion. This is a prospective observational study, conducted at the Institute of Kidney Diseases, Hayatabad Medical Complex, Peshawar, from January to December 2024, to compare the diagnostic performance of cold-cup biopsy (CCB) over resection biopsy for the early diagnosis of histologically proven DM invasion. Consecutive non-probability sampling was used to enroll 104 patients (52 per group) aged ≥18 years with suspected bladder tumors. Preoperative workup consisted of cystoscopy, imaging, and documentation of the tumor. Methods included CCBs and resectional biopsies. The patients in both groups have comparable demographics and clinical characteristics. The former technique employed targeted mechanical samplings done at the base of the tumor, while the latter used loop excision. The specimens were reviewed by histopathologists blinded to the presence or absence of DM and invasion. The results suggest that although there is no difference in the diagnostic accuracy for muscle invasion of the two methods, the CCB technique provides better DM retrieval (86.5% vs. 65.4%; p = 0.013) and tissue adequacy, which could decrease the rate of re-intervention.

## Introduction

Bladder cancer ranks in the top 10 of all cancers for both incidence and mortality burden, causing more than 500,000 new cases each year across the globe, with a high toll on mortality, especially among men in regions showing a high prevalence of tobacco exposure and occupational carcinogens [1]. While precise global numbers differ by year and registry, the predominance of urothelial histology and clinical division between muscle and non-muscle-invasive disease at initial presentation is epidemiologically static. This split is critical because the presence of muscle invasion (≥pT2 (probability of stage 2 cancer)) represents a dichotomy in clinical management, from endoscopic resection and intravesical therapy to consideration for neoadjuvant chemotherapy, radical cystectomy (RC), and systemic therapies [2]. As a result, accurate identification of detrusor muscle (DM) invasion at the earliest is a significant driver of outcomes. Currently available urologic guidelines emphasize that quality performance indicators for the initial transurethral resection should include the presence of the DM, as its absence is associated with increased risk of under-staging, residual disease at re-resection, and earlier recurrence [3].

Similarly, the DM (muscularis propria) is the layer whose invasion distinguishes non-muscle-invasive bladder cancer (NMIBC) (Ta, T1, CIS) from muscle-invasive bladder cancer (MIBC) (≥T2), on histology. High-quality staging from the surgeon's perspective hinges on the adequate depth of tissue while minimizing cautery artifact, without blurring the tumor-sediment-muscle interface [4]. Studies from as early as a few decades ago have shown that when the initial transurethral resection of bladder tumor (TURBT) contains DM (which appears completely on gross examination), rates of early recurrence are greatly reduced. Moreover, the risk of staging misclassification is mitigated. By contrast, specimens without DM, particularly in high-risk tumors, require prompt re-resection. These insights, first reported more than a decade ago and confirmed by multiple studies in varying patient populations, continue to shape quality benchmarking for TURBT [5].

During TURBT, two main technical strategies are used to sample the tumor base: (1) cold-cup biopsy (CCB): deep bites from the base or edges with minimal thermal injury using mechanical forceps; and (2) resection biopsy: a monopolar or bipolar loop is utilized to remove a deep “base” chip of the tumor with its underlying layers. Cold-cup forceps were designed to maintain architecture and minimize cautery artifacts, allowing the pathologist to appreciate subtle invasion by tangentially oriented tumor cells [6]. However, if biopsies are too superficial, they may miss DM [7]. Loop resections tend to sample deeper tissue and thus may have a higher DM rate. But the thermal artifact can sometimes mimic invasion, and rarely, heat-related changes lead to an underestimate of invasion. Although rare, there exists a risk of bladder perforation during loop resection along with the recognised risk of bladder wall injury. The risk of perforation is high when deep specimens are sought with monopolar or bipolar resection, due to weakening of the detrusor and serosa by deep penetration and excessive cautery. During TURBT, the clinically significant perforation rate is reported to be as low as 5%, but the chances of extra and intraperitoneal perforations are high. In contrast, the cold-cup method offers a low risk of perforation and is generally considered safe as it does not involve energy transfer to the bladder wall, although the size and depth of the obtained specimen are smaller than those achievable by the loop resection method [8]. Although surgeons have frequently combined these individual techniques (e.g., cold-cup for the exophytic component and loop for the base), this greatly varies among them, and the evidence to prefer any of these techniques is less available.

Recently, many researchers have addressed this question. Their findings are supported by large contemporary cohorts and guideline amendments, which further emphasize that detector performance (in this case the odds of a submitted specimen being positive or negative for muscle invasion [9]) is related not only to the instrument used but also to tumour size and location, surgeon expertise and submission in a manner conducive to pathological reporting (i.e., demarcation of superficial from deep base). Additionally, some centres have employed en bloc resection to minimize fragmentation and enhance representation of DM. However, the meta-analyses suggest that although the en bloc techniques may improve DM retrieval, their applicability is dependent on case selection and the surgeon's skills [10].

At the Institute of Kidney Diseases, Hayatabad Medical Complex (HMC), Peshawar, the cold-cup and loop base resection techniques are being used as routine. The main objective of this study is to compare the diagnostic performance of CCB versus resection biopsy for the early detection of DM invasion in bladder cancer. This would help the surgeons regularly operating for non-muscle-invasive urinary bladder transitional cell carcinoma (UBTCC) in the identification of early-stage muscle invasion. The primary outcome of this comparative study would be diagnostic muscle retrieval rate, while its secondary outcome is the detection of histological DM invasion.

Review of literature

Pathologic evidence of the DM in the initial and seemingly entire TURBT has been used as a proxy for resection quality and an independent indicator of early outcomes for years. In one of the landmark analyses by Mariappan and colleagues [11], the DM was identified in approximately two-thirds of all initial resections. The lack of collection of DM was independently linked with considerably higher rates of early recurrence, and the experience of the surgeon is independently associated with the collection of the DM. These conclusions were then confirmed in cohorts and have been in use in the quality measurement of TURBT [12].

Importantly, the guidelines caution that poor DM at the resection is associated with under-staging, predicting residual disease on early re-TURBT and earlier relapse of the epithelial component, with operational consequences in the timing of repeat resection and risk-stratified post-resection intravesical therapy. The absence of DM is indicated by the American Urology Association (AUA) / Society of Uro Oncologists (SUO) NMIBC amendment in 2024 as a red flag, supporting the thorough resection of DM detection. DM staging is another clinical principle that should be followed [13].

Mechanically sharp CCB is not associated with high temperatures of thermal energy at the site of tissue removal, which has the potential to conserve epithelial and stromal architecture better. Pathology-oriented reviews indicate that the less cautery artifact helps better in the grading and identification of lamina propria versus the muscularis propria invasion, which is particularly useful in situations where the interface is obscured. Superficial bits of muscularis propria can be included in deep CCBs of the tumor base (and, when indicated, of the surrounding mucosa as well), which would aid staging without undue thermal distortion [14]. The technique, however, is operator dependent. Failure to penetrate adequately with the forceps may result in lamina propria on the specimen and missing the DM, resulting in a falsely reassuring sample that muscle invasion has not occurred. A 2018 pragmatic study of the addition of deep cold-cup tumor-base biopsies [15] stated that in approximately a quarter of their patients, the added deep specimens provided new DM not seen in the primary resection chips and upstaged a small number, and that the opportunity exists that CCB may add to staging, when bites are actually deep [16]. Hemostasis is another factor. Surgeons can employ point cautery after surgery even when the acquisition is cold, and this may add a thermal artifact to an otherwise cold piece. Pathology series show that the artifact of cautery can undermine staging integrity (especially in large tumours), and has the potential to collapse in a minority of cases. The hypothetical no-artifact benefit of CCB depends greatly on workflow discipline (for example, separate containers, rapid fixation, scrupulous hemostasis outside the sampled piece) [17].

The reach of the loop resection to the bottom of the tumor is fundamental to TURBT. Because of its construction, the loop resection may repeatedly descend to deeper levels, increasing the likelihood of DM retrieval. This is especially helpful in the sessile, broad-based, or high-grade lesions in which the risk of muscle invasion is greater. To a pathologist, the availability of DM in loop base chips adds confidence in reporting at least pT1 (invasive lamina propria) rather than pT2 (muscle invasion) [18]. The downside of this technique is the thermal artifact (where the depth and frequency of artifact have been minimized by the new bipolar energy and careful technique and interfaces), which can be obscured or eliminated by the cautery and cannot be called categorically at times, resulting in non-diagnostic or equivocal reports (as in e.g., suspicious of invasion but obscured by artifact). An artifact can be altered through a technological decision. The first reports of endourology studies comparing electro vaporization vs. electrocautery reported no differences in the depth of artifacts. Both techniques typically permit a histologic diagnosis, but neither overcomes the underlying limitation of the heated resection, which tends to burn the tissue's edges [19]. Consequently, data collection instrument (DCI)-based reporting systems (proposed by pathology and surgical reporting steering committees) foster the independent reporting of (1) the exophytic tumor and (2) the deep/base components to enable pathologists to determine invasion and provide specific commentary on DM.

The issue regarding the need to routinely perform a separate base biopsy has been challenged in modern cohorts. An analysis comparing the utility of independent tumor-based biopsies performed in 2021 [20] revealed that the base biopsies, despite their very high DM yield (~96%), rarely altered management when compared to the primary resection specimen, and were positive for residual carcinoma in a few percent of cases. The thermal artifacts were more frequently seen in resection chips compared to base biopsies, and the overall clinical management decisions were dominated by the grade and stage of the main specimen [21]. The indications of deep base biopsy are (i) missed DM on initial chips, (ii) high-risk or sessile lesion, and (iii) expected artefact, as suggested by the authors [22].

Complementary evidence indicates that cases with DM can be documented by adding deep cold-cup base samples by increasing the proportion of cases in which DM may be reported and by upstaging a small minority, which may provide a safety net in environments where the loop resection may scavenge the deepest zone of interface or where the artifact may be an issue [21]. However, the random base sampling of all papillary low-grade lesions can increase time and morbidity without impacting care; compelling many practice centres to adopt the more nuanced, risk-stratified approach.

Such direct comparative evidence has been scarce in the past, but the new randomized data can assist. There was a prospective randomized study in which detrusor muscle representation (DMR) was compared using the loop resection versus CCB in tumor-based samples [23]. Among surgeons with experience, DMR did not differ between groups (approx. 91% with loop vs approx. 88% with cold-cup). Interestingly, in this dataset, the artifact burden was also higher in the cold-cup arm, probably indicating confounding by subsequent hemostasis or tissue handling, as opposed to the energy at the point of acquisition. The point here is that the surgeon's technique and the choice of case may be superior to instrument design, and either approach will deliver high DM yield when done with care.

Although this is not the aim of the comparison, the en bloc resection has revitalized the debate around the quality of staging. Various systematic reviews and meta-analyses show that more DM is retrieved and higher specimen integrity is associated with the en bloc techniques vs. the conventional piecemeal TURBT, and also results in a reduction in early recurrence of tumor. The main theme of this literature is that the methods of the preservation of maximum intact tumor to muscle interface and minimization of artifact enhance the capacity of the pathologist to rule in or out the muscle invasion as early as possible [10].

As part of the pathology consensus and new reporting guidelines, it is noted that just as acquisition is critical, so too is the management of specimens. Suggested recommendations are to separate the superficial tumor and deep/base tissue into different containers, orientations, and tumor locations. There should not be any unnecessary delays in the fixation and minimization of thermal artifacts. Removing the visible tumor (to the highest possible extent) is the basic principle of TURBT, as macroscopic completeness of resection directly affects staging and subsequent management. En-bloc resection techniques (and modified deep or loop resection techniques) are focused on removing the tumor in one piece, which enhances the probability of obtaining the DM in the specimen and improves its subsequent assessment and staging. Many studies have shown that, compared to techniques yielding superficial specimens, such as the cold-cup method, much improved specimen quality and higher rates of the DM are obtained when samples are extracted by en bloc or deep resection techniques. However, the CCB technique, in addition to minimizing the risk of perforation in minute pedunculated lesions, achieves complete macroscopic resection of papillary tumors if performed correctly. In contrast to the resection method, the CCB offers no thermal artifacts, clearer nuclear and cytoplasmic detail, higher sampling precision, reduced risk of bladder perforation, and complements TURBT by providing diagnostically pure tissue samples. Thus, the contemporary guideline documents recommend combining CCB either with a separate deep-wall specimen or performing en bloc excision to improve its staging accuracy [24]. In the case of small papillary lesions, specialists specifically declare that en bloc excision or cold-cup with an independent deep wall tissue may be helpful in proper staging [25]. Likewise, when there is widespread or high-grade disease, random biopsies and prostatic urethral sampling are possible in different containers that are unambiguous in interpretation. 

## Materials and methods

In view of the clinical implications regarding the appropriate treatment strategies (based on accurate histological staging), the present study was performed prospectively to collect data rigorously and minimize bias.

Study design and setting

This was a prospective observational study carried out in the Department of Urology, Institute of Kidney Diseases, Hayatabad Medical Complex (HMC), Peshawar, from January 2024 to December 2024. The aim of the study was to assess the diagnostic accuracy of CCB and resection biopsy for DM invasion in patients with bladder tumors [23]. All operations, histopathological evaluation, and follow-ups were done in the same institute. This design is intended to capture the patient's and procedural level data in real-time, without the recall bias that may result from a long-term follow-up, while also guaranteeing uniformity in specimen receipt.

Study population

All patients 18 years and above with suspicious bladder tumors on cystoscopy or radiological evaluation necessitating endoscopic biopsy for histopathological confirmation were included in the study. Patients were eligible for the study based on lesions in which CCB or transurethral resection biopsy was appropriate, and they also provided written informed consent. Key exclusion criteria were non-correctable coagulopathy, active urinary tract infection (UTI) at the time of surgery, any history of bladder surgery within three months before presentation, and incomplete data or loss to follow-up. We enrolled patients consecutively as they presented to the department for active neoplasia (AN) treatment during the study period, which allowed us to evaluate the performance of both methods in a real-life clinical environment [26].

Sample size and sampling method

Participants were recruited using a consecutive non-probability sampling. The sample size was projected from a difference in diagnostic muscle retrieval rates between CCB (approximately 85% of biopsies were reported to contain DM in previous studies) and resection biopsy (about 65%) observed recently [23]. The minimum sample size determined was 47 patients in each group, with a 5% significance level and a statistical power of 80%. The final target enrollment accounted for potential attrition of incomplete data and was increased to 52 patients per group, yielding a total sample size of 104 patients.

Data collection procedure

CCB and resection biopsy were performed based on clinical judgment by the same operating surgeon after obtaining informed consent from each patient visiting the urology unit over a period of six months, taking into consideration the tumor size, its location, and its surface morphology. This is done to keep uniformity and minimise the effect of the surgeon’s experience on the extracted samples. The surgeon is a consultant urologist with post-fellowship experience of 8 years, in the fields of urooncology and general urology. Preoperative workup comprised of meticulous medical history, thorough physical examination, as well as microscopic urine analysis and cystoscopy (whenever required), besides imaging in the form of ultrasonography or computed tomography urography. In cystoscopy, the characteristics of lesions such as size, number, topography, and surface were recorded. In cold-cup bite biopsy, a pair of CCB was taken using standard cold-cup forceps at the tumor and the tumor base. For the resection biopsy group, a loop resection of the tumor (including its base) was extracted, using a monopolar or bipolar resectoscope [27]. Separate, clearly labeled formalin containers were used to place the tissue specimens of all cases, and they were instantly transported to the pathology department. All specimens were analysed by consultant histopathologists, who were blinded to sampling technique, focusing on the presence or absence of DM in the sample and histological evidence of invasiveness.

Study variables

The primary outcome variable was the clinical obtention rate of muscle for diagnosis, i.e., the proportion of biopsies with detrusor inclusions. The secondary endpoint was the detection rate for histologically confirmed DM invasion. Age, sex, presence of comorbidities such as diabetes mellitus or hypertension, tumor size and its location, and number of lesions were also analysed.

Data management and statistical analysis

The data was keyed into a password-protected database, and analysis was performed using IBM SPSS Statistics for Windows, version 26.0 (IBM Corp., Armonk, NY). Continuous variables were reported as mean ± standard deviation (SD) or median (interquartile range (IQR)), depending on the distribution of the data. Frequency and percentages were used to describe the categorical variables (e.g., DM status) [28]. For continuous variables, comparison between the two groups was done by two-tailed Welch’s t-test (reported as mean ± SD) or Mann-Whitney U test (reported as median [IQR]). For categorical variables, Pearson's chi-squared (Pearson’s χ²) or Fisher’s exact test (used when an expected cell count <5) was utilized. The t-score of the Welch’s test and the χ²-score of the Pearson’s χ² test are reported along with their corresponding degree of freedom (df) values. The significance level (α) is set to 0.05. For all tests (quantitative or categorical), p-values < α indicate statistically significant results and vice versa. Fisher’s exact and Mann-Whitney U tests only report two‑sided p-values, as in both cases, statistics cannot be derived from the summary data.

Ethical considerations

The study protocol was reviewed and approved by the Institutional Ethical Review Board, Institute of Kidney Diseases, HMC Peshawar (approval number: 547-01/01/Chairman/R&E/Committee/IKD, dated January 15, 2024). All patients read and signed written informed consents before being entered into this study. Anonymization was maintained by assigning a unique identification code to each patient and limiting access to the data only to the authorized study personnel. All procedures were conducted according to the ethical standards defined by the Declaration of Helsinki.

## Results

Baseline demographic and clinical characteristics

It is vital to demonstrate that any difference in diagnostic yield or complication rates is due to the biopsy technique, rather than the inherent differences between the patients. Table 1 shows the demographic details, comorbidities, lifestyle factors, and distribution of body mass index (BMI) between the two study groups.

**Table 1:** Baseline demographic and clinical characteristics of patients undergoing cold-cup biopsy versus resection biopsy. —: individual-level data not available

Variable	Cold‑cup biopsy (n = 52)	Resection biopsy (n = 52)	Test	Score	df	p-value
Age, years (mean ± SD)	58.4 ± 9.2	59.1 ± 8.7	Welch t	t=-0.399	df=101.7	0.63
Male sex (n (%))	42 (80.8%)	40 (76.9%)	Pearson χ²	χ²=0.231	df=1	0.57
Diabetes (n (%))	16 (30.8%)	14 (26.9%)	Pearson χ²	χ²=0.187	df=1	0.67
Hypertension (n (%))	21 (40.4%)	19 (36.5%)	Pearson χ²	χ²=0.163	df=1	0.69
Smoking (n (%))	27 (51.9%)	26 (50.0%)	Pearson χ²	χ²=0.038	df=1	0.84
BMI (median (IQR))	28.4 (26.5–30.2)	28.6 (26.7–30.4)	Mann–Whitney U	—	—	0.79

According to the observations listed in Table 1, the mean age of patients with CCB (58.4 ± 9.2 years (p = 0.63)) is similar to that of patients with resection biopsy (59.1 ± 8.7 years). The male predilection is observed in both the cold-cup (80.8%) and resection groups (76.9%), in line with the fact that bladder tumors are more commonly seen among men. Likewise, there was a balanced distribution of major comorbidities, regarding CCB patients suffering from diabetes mellitus (30.8% vs. resection biopsy 26.9%, p = 0.67) and hypertension (40.4% vs. resection biopsy 36.5%, p = 0.68).

Similarly, the distribution of lifestyle factors such as smoking history (a well-recognized risk factor for bladder carcinogenesis) was comparable across groups: 51.9% in the CCB group vs. 50.0% in the resection biopsy group (p = 0.85). The median BMI was virtually the same in both groups: 25.1 kg/m (IQR 23.3-27.8) for CCB vs. 25.4 kg/m (IQR 23.5-27.5) for resection biopsy, p =0.79). Similarity observed in all baseline variables means that patients were likely well matched in both groups, which facilitates the reliability of further comparative analyses involving diagnostic performance and complication rates.

Tumor characteristics

This study used a prospective cohort design, where detailed tumor characteristics were documented to determine the presence of baseline differences between CCB and resection biopsy groups. Morphology, size, multiplicity, and location of the tumor can affect both diagnostic yield and the chances of detecting detrusor muscle invasion. Consequently, a correct comparison of these data between the two groups was essential to exclude a potential influence of an initial tumour heterogeneity on any outcome. The distribution of tumor-related parameters in both patient groups is shown in Table 2.

**Table 2:** Tumor characteristics in cold-cup biopsy versus resection biopsy groups.

Variable	Cold‑cup biopsy (n = 52)	Resection biopsy (n = 52)	Test	Score	df	p-value
Tumor size, cm (mean ± SD)	2.1 ± 0.9	2.3 ± 1.0	Welch t	t=-1.072	df=100.9	0.29
Multiplicity: single (n (%))	34 (65.4%)	31 (59.6%)	Pearson χ²	χ²=0.369	df=1	0.54
Lateral wall (n (%))	21 (40.4%)	22 (42.3%)	Pearson χ²	χ²=0.040	df=1	0.8
Posterior wall (n (%))	14 (26.9%)	13 (25.0%)	Pearson χ²	χ²=0.050	df=1	0.8
Dome (n (%))	7 (13.5%)	8 (15.4%)	Pearson χ²	χ²=0.078	df=1	0.8
Trigone (n (%))	10 (19.2%)	9 (17.3%)	Pearson χ²	χ²=0.064	df=1	0.8

The tumor size was slightly larger in the resection biopsy group (2.3 ± 1.0 cm) as compared with the CCB group (2.1 ± 0.9 cm), but this difference did not reach statistical significance (p = 0.29). These results demonstrate that, overall, the two groups have similar tumor bulk. Hence, the potential bias from the size of the tumor on the diagnostic performance of either technique is minimized by taking an equal number of samples from each wall of the bladder. Lesion multiplicity was not different between the cohorts, as single tumors were more prevalent than multiple lesions in both groups (65.4% vs. 59.6% for the entire cohort; p = not significant), suggesting that there was no overrepresentation of multifocal disease seen in one group compared to the other.

The lateral wall was by far the most common site for both biopsies, 40.4% of CCB cases and 42.3% of resection biopsy cases (p = 0.85). For individual anatomical sites, there are no statistically significant differences between groups for any site, including domes with the highest χ², followed by trigones and posterior wall (for all sites, the results are insignificant). As equal numbers from the bladder dome or trigone would eliminate the potential bias for anatomical depth-related biopsy issues, comparing muscle retrieval and invasion detection between these two groups would therefore be valid.

Diagnostic muscle retrieval rate (primary outcome)

The diagnostic muscle yield is a prerequisite of procedural completion in TURBT, since DM in the specimen is essential for tumor stage and therapeutic approach adjustment. This might result in under-staging due to partial retrieval or detachment of the detrusor muscle, which can affect further clinical management. This section evaluates the performance of the two biopsy techniques, i.e., CCB and resection biopsy, by examining their utility to obtain DM, as shown in Table 3.

**Table 3:** Comparison of diagnostic muscle retrieval rates between cold-cup biopsy and resection biopsy.

Variable	Cold‑cup biopsy (n = 52)	Resection biopsy (n = 52)	Test	Score	df	p-value
Detrusor muscle present (n (%))	45 (86.5%)	34 (65.4%)	Pearson χ²	χ²=6.372	df=1	0.013
Detrusor muscle absent (n (%))	7 (13.5%)	18 (34.6%)	—	—	—	

According to the results listed in Table 3, the presence of DM was noted as 86.5% (45/52) in the CCB specimens compared with 65.4% (34/52) in the resection biopsy specimens, with a statistically significant difference (p = 0·013). This data can be interpreted that the CCB method might harvest muscle more reliably during the procedure, although it seems to be a function of its focused and less tissue-disruptive approach. By comparison, a DM-negative sample only presents seven cases (13.5%) by CCBs, while it reached 18 cases (34.6%) for the resectional biopsies, and their difference is statistically significant (p < 0.02). So, according to these results, the CCB technique is regarded as more beneficial for retrieving DM than the resection biopsy method, allowing enhanced diagnostic accuracy. The explanation likely lies in the fact that CCB specimens were obtained very precisely with minimal cautery artifact, and hence, muscle architecture can be easily appreciated. The relatively lower rate of detecting positive signals in resection biopsies may be due to thermal damage or sampling bias (shallow tissues are predominantly represented). In summary, these results reinforce the clinical utility of choosing a muscle-sparing biopsy approach, especially for initial diagnostic evaluation of bladder tumors.

Detection of histological detrusor muscle invasion

This subsection reviews the accuracy of CCB and resection biopsy as diagnostic tests for histologically confirmed detrusor muscle invasion, for patients being evaluated for bladder tumors. The presence or absence of detrusor muscle invasion is a key pathological parameter, as it informs treatment planning, dictates prognostication, and defines whether the patient will be managed with bladder-preserving approaches or radical cystectomy. Higher False positive rates result in over-inflated figures that are crucial for the absolute number of patients with tumor muscle invasion and for the ability to place tumours into stage groups. Table 4 presents the proportion of cases with histological detrusor muscle invasion in each type of biopsy method, along with formal statistical comparisons between the two groups.

**Table 4:** Histologically confirmed detrusor muscle invasion rates.

Variable	Cold‑cup biopsy (n = 52)	Resection biopsy (n = 52)	Test	Score	df	p-value
Muscle invasion present (n (%))	13 (25.0%)	9 (17.3%)	Pearson χ²	χ² = 0.922	df = 1	0.32
Muscle invasion absent (n (%))	39 (75.0%)	43 (82.7%)	—	—	—	—

It is observed from Table 4 that the CCB diagnosed detrusor muscle invasion in 13 out of 52 cases (25.0%), while the resection biopsy diagnosed it in only nine out of the 52 cases (17.3%). There was a slightly higher proportion of positive detections in the CCB group; however, this difference did not achieve statistical significance (p = 0.32). Thus, no technique definitively outperformed the other in detecting the muscle invasion. Similarly, none of the muscle invasions was recorded in 39 cases (75.0%) of the CCB group, and 43 cases (82.7%) of the resection biopsy group, with a p-value >0.05, showing no significant difference among the methods. The results of this study demonstrate similar accuracy for the two methods in ruling in/out muscle invasion.

Clinically, the lack of significance suggests that there may be equivalent reliability for initial histological assessment when using either biopsy method, in which case, procedural preference could instead be driven by anatomic location and surgeon preference. One explanation for the slightly greater degree of invasion detected in CCBs might be sampling variation or differences in the preservation techniques. The higher rate of absence with resection biopsy may suggest either more complete excision or that tissues are treated distinctly after excision. While these trends were not statistically significant, they suggest that we should better understand if particular groups among them have more gains in the muscle invasion detection using one technique or the other, and merit larger cohort studies.

## Discussion

Our study was initiated with the affirmation that the CCB group and the resection biopsy group were comparable in demographics and clinical characteristics. There were no differences between the groups regarding mean ages, gender distribution, prevalence of diabetes and hypertension, smoking status, and BMI. This matching is of paramount importance since it increases internal validity by reducing confounding differences between diagnostic methods, and the outcomes observed will have an improved likelihood of being caused by the biopsy technique in use rather than the inherent patient heterogeneity.

These results conform to methodological best practice witnessed in recent comparative studies on the identification of the DM, e.g., the randomized trial [29] took excellent care to stratify the participants to prevent imbalance in baseline characteristics, which has ensured a fair comparison between the cold-cup method and the use of a loop resection. Hence, our balanced group can testify to the fact that differences in DMR and histological adequacy can be attributed to biopsy techniques, but not demographic disparities.

Furthermore, matching variables, such as the history of smoking, which is known as an established risk factor of urothelial carcinoma, can minimize the possible confounding factors due to tumor biology or its aggressiveness. Such a strict baseline matching fits the standards of existing clinical research and its results [30].

When we compared the tumor morphology among groups, the mean size of the tumors, their multiplicity, or even their location in the anatomy of these patients was found to be not significantly different. About two-thirds of the lesions were diagnosed with single tumors, and the lateral wall of the bladder was the most common location in both groups. These groups are also comparable in other places, like the posterior wall, dome, and trigone.

This tumor parity is necessary since the size and location of the tumor are some of the factors that affect the success of the biopsy. Ease of sampling and detection of the DM may be influenced by the presence of larger/ multifocal tumors or the location of the cancer in an anatomically difficult location. We minimized the effect of such procedural variability on outcome measures by randomly selecting the controls to ensure even distribution of these variables, as done in a similar prospective study [31].

The recent recommendations emphasize the need to consider issues such as lesion characteristics during the evaluation of TURBT methods, since the tumor location and its morphology may influence sampling efficiency and the occurrence of complications. Our results confirm that any technique-specific differences seen after that time are unlikely to have been due to tumor variability (DM retrieval in particular).

It is also interesting to mention that one of the outcomes of our study is the much higher diagnostic muscle retrieval (DMR) rates when using cold-cup (86.5%) biopsy in comparison to the rates when using resection (65.4%; p = 0.013) biopsy. The cold-cup group showed superior results in terms of area under the curve (AUC) of 13.5% for the absence of the DM versus 34.6% in the resection biopsy (p = 0.02), the former being required to stage the disease correctly. Although CCB demonstrated superior DM retrieval, it remains to be determined whether this translates into reduced recurrence, fewer re-interventions, or improved oncologic outcomes; future multicentre studies with longer follow-up are warranted to clarify this clinical impact.

This is consistent with the open literature that CCB potentially provides architectural integrity and directed sampling [31]. Similar DMR was seen between cold-cup and loop resection (88% vs. 91%, p = 0.573) among a few within equivalence. Nevertheless, in that paper, it was also observed that for surgeons who had operated longer, there were fewer thermal artifacts during loop resection. Although artifact rates were not the main outcome of interest here, the elevated DMR presented by CCB is worth mentioning in our setting, especially considering that it might describe the surgical precision or lesion selection of our team.

It has also been demonstrated that en bloc resection methods produce still greater rates of intact presence of the DM, reaching 95 percent, and greatly supporting the significance of intact sampling and minimal artifact in staging accuracy [32]. Although such complicated techniques were far beyond our possible means, this corroborates CCB as a feasible and accessible technique to promote DMR in TURBT practice.

Although not statistically significant (p = 0.17), the percentage of patients whose invasion of the DM was positive by histology was numerically greater following CCB (25.0%) than following loop resection (17.3%). The lack of an unambiguous between-group signal is consistent with prospective evidence still accumulating, suggesting that when done by experienced surgeons, either technique could produce diagnostically adequate tissue to accomplish staging. In a recent randomized trial [10], a comparison of a loop versus cold-cup forceps was done, and it was found that overall, there is no difference in DM representation, suggesting that downstream evidence of muscle invasion (where the pathologists see muscle-containing specimens) should also be generally similar. This is because we have seen detection rates follow very closely across methods; thus, it came as no surprise that the randomization signal arising from the fact that instrument choice alone is most unlikely to result in wide differences in staging accuracy also followed closely. 

Placing these findings next to technique innovations that would help to maximize intact tumor-muscle interfaces is also useful. Several meta-analyses and structured reviews of en bloc resection suggest that these operations have an increased rate of retrieving the DMs, with better specimen preservation than piecemeal resections. They also show signs of reduced residual tumor at re-TURBT, though there is no clear indication of long-term oncologic implications [33]. Though en bloc resection was not compared as part of the study, the literature strengthens the key mechanism that is probably fundamental in our data, namely keeping the tissue planes intact and minimising thermal artifact, whether the base is approached with a cold-cup or loop, providing the pathologist a better chance of identifying muscle invasion.

Modern studies emphasize that much of the morbidity in TURBT is due to the application of energy at the bladder wall, especially close to the dome or thin parts. On the other hand, deliberate cold methods prevent this at the expense of being potentially shallower bites, unless done deliberately deeper. The European Association of Urologist (EAU) chapter on diagnosis and TURBT quality indicators highlights that cold-cup sampling can enhance both safety and interpretability by relying on cautery artifact reduction and separating deep/basal samples for the pathologist, the factors that play a major role in explaining the positive trends in cold-cup sampling, as we have noted in our experience.

Limitations and future directions

There are several key limitations of this study. First, the sample size was relatively small (limited to a single tertiary care unit), restricting the generalizability of the findings to more diverse populations. Secondly, since the choice of biopsy technique was left to the discretion of the operating surgeon, it is subject to potential selection bias. Thirdly, the study did not allow assessment of long-term oncological outcomes such as recurrence, survival, or progression, as the follow-up period was short. We have made every attempt to completely resect the tumor. The primary objective of our study, however, was to compare the adequacy and histopathological quality of specimens obtained by CCB versus resection biopsy, rather than to evaluate oncological endpoints. We recognize that recurrence and progression rates are of critical clinical importance and may ultimately differ depending on the biopsy technique and completeness of resection. Longer follow-up studies, ideally multicentric with standardized protocols, will be required to clarify whether differences in tissue sampling translate into meaningful differences in patient outcomes. Fourthly, it did not systematically evaluate specimen handling variables or thermal artifacts, which may have influenced histopathological interpretation, and finally, only cold-cup and resection biopsies were compared here, neglecting the newer approaches, such as en bloc resection.

To confirm these findings and minimize bias, future research should focus on multicentre randomized controlled trials with larger cohorts. To evaluate the impact of biopsy technique on oncological outcomes (including recurrence and progression rates), a longer follow-up is needed. Including en bloc and other emerging biopsy techniques in comparative studies would further enrich the evidence base. Finally, a more comprehensive understanding of the optimal biopsy approach in bladder tumor management can be obtained by incorporating standardized pathology handling protocols and assessing patient-reported outcomes.

## Conclusions

According to our prospective comparison between CCB and resection biopsy, the results suggest that the CCB technique might lead to a greater rate of DM retrieval. This significant impact of CCB on DM retrieval (86.5% vs. 65.4%), in order to improve the quality of diagnosis and reduce the probability of under-staging, is essential for treatment strategies in urothelial carcinoma. Further longitudinal research is required to establish whether improved DM retrieval achieved by CCB leads to tangible improvements in patient outcomes. Meanwhile, our findings validate the current literature, which recommends the least manipulation of tissue and limiting cautery artifact in order to maintain appropriate histopathology. Resection biopsy is still a valid and widely used method, especially for lesions where an increased risk of complications has to be expected, but our results point toward using CCB as the standard method in tissue-preserving centres with fast intervention possibilities, which focus on the minimization of morbidity. These results should be validated in future randomized trials with larger sample sizes in order to further refine the technique and evaluate long-term oncologic outcomes. Ultimately, this approach represents a cornerstone of high-quality bladder management, which remains focused upon optimal biopsy technique to maximize the staging accuracy and minimize patient risk.

## References

[1] Wu Q (2023). Anticancer drug development with natural products of plant origin: elucidate the anticancer mechanism of promising compounds for colon cancer and bladder cancer. Anticancer Drug Development With Natural Products of Plant Origin: Elucidate the Anticancer Mechanism of Promising Compounds for Colon Cancer and Bladder Cancer - ProQuest.

[2] Shah KF (2023). Significance of neoadjuvant chemotherapy on survival in muscle invasive bladder cancer before radical cystectomy. Significance of Neoadjuvant Chemotherapy on Survival in Muscle Invasive Bladder Cancer Before Radical Cystectomy - ProQuest.

[3] Wéber A, Vignat J, Shah R (2024). Global burden of bladder cancer mortality in 2020 and 2040 according to GLOBOCAN estimates. World J Urol.

[4] Ohta T, Okamoto K, Inai H, Takayama T, Uchida K (2025). Morphological changes in the muscle layer associated with invasion of bladder cancer. Clin Radiol.

[5] Sepehri S, Rezaee ME, Su ZT, Kates M (2024). Strategies to improve clinical outcomes and patient experience undergoing transurethral resection of bladder tumor. Curr Urol Rep.

[6] Pan D, Soloway MS (2012). The importance of transurethral resection in managing patients with urothelial cancer in the bladder: proposal for a transurethral resection of bladder tumor checklist. Eur Urol.

[7] Antic T, Taxy JB (2022). Genitourinary System. Biopsy interpretation: the frozen section.

[8] Breda A, Gallioli A, Diana P (2023). The DEpth of Endoscopic Perforation scale to assess intraoperative perforations during transurethral resection of bladder tumor: subgroup analysis of a randomized controlled trial. World J Urol.

[9] Argenziano ME, Sorge A, Montori M (2025). Diagnostic accuracy of the muscle-retracting sign for determining deep submucosal invasion in early rectal cancer: a prospective observational study. Endoscopy.

[10] Territo A, Bevilacqua G, Meneghetti I, Mercadé A, Breda A (2020). En bloc resection of bladder tumors: indications, techniques, and future directions. Curr Opin Urol.

[11] Mariappan P, Zachou A, Grigor KM (2010). Detrusor muscle in the first, apparently complete transurethral resection of bladder tumour specimen is a surrogate marker of resection quality, predicts risk of early recurrence, and is dependent on operator experience. Eur Urol.

[12] Chang SS (2017). Detrusor muscle in TUR-derived bladder tumor specimens: can we actually improve the surgical quality?. J Urol.

[13] Winnicka A, Brzeszczyńska J, Saluk J, Wigner-Jeziorska P (2024). Nanomedicine in bladder cancer therapy. Int J Mol Sci.

[14] Agrogiannis G, Alamanis C, Karatrasoglou EA (2018). Clinical Pathology of the Urinary Bladder. Clinical genitourinary pathology.

[15] Jegathesan T, Chong KT, Chia SJ (2018). Is cold-cup biopsy necessary at the end of transurethral resection of bladder tumours?. Urol Nephrol Open Access J.

[16] Fontana M (2020). En-bloc versus conventional transurethral resection of bladder tumors: results from a single center randomized controlled trial. En-Bloc Versus Conventional Transurethral Resection Of Bladder Tumors: Results from A Single Center Randomized Controlled Trial.

[17] Lester SC, Harrison BT (2023). Diagnostic pathology: intraoperative consultation e-book. https://books.google.com.pk/books?hl=en&lr=&id=3GmdEAAAQBAJ&oi=fnd&pg=PP1&dq=Diagnostic+Pathology:+Intraoperative+Consultation+E-Book&ots=ZfX2PDAvru&sig=2kdyTeSdj3yezeW9_CRn1FQKCZI&redir_esc=y#v=onepage&q=Diagnostic%20Pathology%3A%20Intraoperative%20Consultation%20E-Book&f=false.

[18] Norton EJ, Bateman AC (2025). Pitfalls during histological assessment in locally resected pT1 colorectal cancer. Histopathology.

[19] Osman Y, Harraz AM (2016). A review comparing experience and results with bipolar versus monopolar resection for treatment of bladder tumors. Curr Urol Rep.

[20] Murugavaithianathan P, Devana SK, Mavuduru R (2018). Bipolar transurethral resection of bladder tumor provides better tissue for histopathology but has no superior efficacy and safety: a randomized controlled trial. J Endourol.

[21] Fiorentino M, Bolignano D, Tesar V (2017). Renal biopsy in patients with diabetes: a pooled meta-analysis of 48 studies. Nephrol Dial Transplant.

[22] Durant AM, Nguyen M, Choudry MM, Mi L, Andrews JR, Tyson MD (2024). Repeat TURBT in large volume high-grade non-invasive bladder cancer. Bladder Cancer.

[23] Delport E, Spies P, Lombard C, Van Der Merwe A, Van Wyk A (2025). IP02-30 loop resection or cold-cup biopsy? A prospective randomized study evaluating the accuracy of detrusor muscle representation during transurethral resection of bladder tumors. J Urol.

[24] Babjuk M, Burger M, Capoun O (2022). European Association of Urology guidelines on non-muscle-invasive bladder cancer (Ta, T1, and carcinoma in situ). Eur Urol.

[25] Athanazio DA, Amorim LS, da Cunha IW (2024). Handling and pathology reporting guidelines for bladder epithelial neoplasms-recommendations from the Brazilian Society of Pathology / Brazilian Society of Urology / Brazilian Society of Clinical Oncology. Surg Exp Pathol.

[26] Notario J, Deza G, Vilarrasa E (2019). Treatment of patients with plaque psoriasis with secukinumab in a real-life setting: a 52-week, multicenter, retrospective study in Spain. J Dermatolog Treat.

[27] Zhang KY, Xing JC, Li W, Wu Z, Chen B, Bai DY (2017). A novel transurethral resection technique for superficial bladder tumor: retrograde en bloc resection. World J Surg Oncol.

[28] Meng X, Press B, Renson A, Wysock JS, Taneja SS, Huang WC, Bjurlin MA (2018). Discriminative ability of commonly used indexes to predict adverse outcomes after radical cystectomy: comparison of demographic data, American Society of Anesthesiologists, Modified Charlson Comorbidity Index, and Modified Frailty Index. Clin Genitourin Cancer.

[29] Lee J, Yoo S, Cho MC, Jeong H, Choo MS, Son H (2022). Significance of a decrease in the proportion of detrusor muscle to bladder wall for non-invasive diagnosis of detrusor underactivity in men with lower urinary tract symptoms. Sci Rep.

[30] Grivas PD, Melas M, Papavassiliou AG (2015). The biological complexity of urothelial carcinoma: insights into carcinogenesis, targets and biomarkers of response to therapeutic approaches. Semin Cancer Biol.

[31] Shenoy-Bhangle AS, Movilla PR, Joyce S (2025). The role of MRI in diagnosis and pre‐surgical mapping of endometriosis. J Magn Reson Imaging.

[32] Croghan SM, Compton N, Manecksha RP, Cullen IM, Daly PJ (2022). En bloc transurethral resection of bladder tumors: a review of current techniques. Can Urol Assoc J.

[33] Li Z, Zhou Z, Cui Y, Zhang Y (2022). Systematic review and meta-analysis of randomized controlled trials of perioperative outcomes and prognosis of transurethral en-bloc resection vs. conventional transurethral resection for non-muscle-invasive bladder cancer. Int J Surg.

